# The Key Roles of PTEN in T-Cell Acute Lymphoblastic Leukemia Development, Progression, and Therapeutic Response

**DOI:** 10.3390/cancers11050629

**Published:** 2019-05-06

**Authors:** Alberto M. Martelli, Francesca Paganelli, Antonietta Fazio, Chiara Bazzichetto, Fabiana Conciatori, James A. McCubrey

**Affiliations:** 1 Department of Biomedical and Neuromotor Sciences, University of Bologna, 40126 Bologna, Italy; alberto.martelli@unibo.it (A.M.M.); francesca.paganell15@studio.unibo.it (F.P.); antonietta.fazio@studio.unibo.it (A.F.); 2 Medical Oncology 1, IRCCS Regina Elena National Cancer Institute, 00144 Rome, Italy; chiara.bazzichetto@ifo.gov.it (C.B.); fabiana.conciatori@ifo.gov.it (F.C.); 3 Department of Microbiology & Immunology, Brody School of Medicine, East Carolina University, Greenville, NC 27834, USA

**Keywords:** lipid phosphatase, PI3K/Akt/mTOR, genetic anomalies, targeted therapy, prognosis

## Abstract

T-cell acute lymphoblastic leukemia (T-ALL) is an aggressive blood cancer that comprises 10–15% of pediatric and ~25% of adult ALL cases. Although the curative rates have significantly improved over the past 10 years, especially in pediatric patients, T-ALL remains a challenge from a therapeutic point of view, due to the high number of early relapses that are for the most part resistant to further treatment. Considerable advances in the understanding of the genes, signaling networks, and mechanisms that play crucial roles in the pathobiology of T-ALL have led to the identification of the key drivers of the disease, thereby paving the way for new therapeutic approaches. PTEN is critical to prevent the malignant transformation of T-cells. However, its expression and functions are altered in human T-ALL. *PTEN* is frequently deleted or mutated, while PTEN protein is often phosphorylated and functionally inactivated by casein kinase 2. Different murine knockout models recapitulating the development of T-ALL have demonstrated that PTEN abnormalities are at the hub of an intricate oncogenic network sustaining and driving leukemia development by activating several signaling cascades associated with drug-resistance and poor outcome. These aspects and their possible therapeutic implications are highlighted in this review.

## 1. Introduction

T-cell acute lymphoblastic leukemia (T-ALL) is an aggressive blood malignant disorder that represents ~25% of acute lymphoblastic leukemia cases in adults [1] and 10–15% in children [2]. Thanks to the use of an intensive combination chemotherapy and modulation of treatment tailored on patient response, the overall survival at five years from diagnosis in pediatric patients has improved to ~80% [3]. However, adults younger than 60 years have five-year survival rates of only 40–50%, whereas older patients display an even worse outcome [4]. Furthermore, among children who relapse and develop refractory leukemia, only about 20% can be successfully treated with currently-available salvage treatments [5]. Moreover, especially children who survive T-ALL are at increased risk of developing secondary neoplasias and other health problems due to the use of high-intensity genotoxic chemotherapy [6]. Hence, more efficacious and less toxic novel treatments are needed for improving the outcome of T-ALL patients and their quality of life, both during and after polychemotherapy.

From a genetic point of view, T-ALL is a very heterogeneous disorder caused by the accumulation of mutations that combine with altered expression of transcription factors during the development of T-cells, thereby leading to aberrant activation of several signaling pathways [7,8]. The analysis of gene expression profiles has allowed the identification of three major subgroups of T-ALL. These subgroups display gene expression patterns closely related to those detectable during thymocyte differentiation [8]. Early T-cell precursor T-ALL (ETP T-ALL) has a gene expression profile typical of immature T-cell precursors, very similar to hematopoietic stem cells (HSCs)/myeloid progenitors. Remarkably, ETP-ALL patients display a pattern of genetic anomalies overlapping with that of acute myeloid leukemia, including Fms-related tyrosine kinase 3 (FLT3) mutations [9]. Early cortical thymocyte T-ALL is usually associated with chromosomal translocations that lead to the aberrant expression of nuclear receptor subfamily 2 group E member (TLX) 1/3 and the related homeobox (HOX) transcription factor family of oncogenes [7]. Late cortical T-ALL is the largest subgroup, accounting for 40–60% of all cases, and typically overexpresses the transcription factor oncogene T-cell acute lymphocytic leukemia 1 (TAL1) with either LIM domain only (LMO) 1 or LMO2 [10].

The phosphatase and tensin homolog, deleted on chromosome TEN (PTEN), is one of the most frequently-mutated/functionally-inactivated oncosuppressors in cancer [11]. PTEN function is lost in a wide variety of human cancers (breast, thyroid, colorectal, endometrial, lung, bladder, melanoma) through somatic mutations, gene silencing, epigenetic alterations, and post-translational modifications that include phosphorylation, oxidation, acetylation, and ubiquitination [12]. PTEN is an inositol lipid phosphatase that dephosphorylates phosphatidylinositol (3,4,5)P3 (PIP3) to yield phosphatidylinositol(4,5)P2 (PIP2). Therefore, PTEN acts as the main negative regulator of the phosphatidylinositol-3 kinase (PI3K)/Akt/mechanistic target of rapamycin (mTOR) signaling network [13]. This cascade plays key roles in the control of a wide range of processes that include cell proliferation, survival, growth, motility, metabolism, and angiogenesis [14]. In general, PI3K has per se a weak driver oncogenic activity, although its upregulation is permissive for growth factor signals via cooperation with other signaling pathways, such as the more potent rat sarcoma (RAS)/rapidly-accelerated fibrosarcoma (RAF)/mitogen-activated protein kinase kinase (MEK)/extracellular signal-regulated kinase (ERK) module [15]. However, in genetically-engineered murine models, several studies have documented a causal role of mutated PI3K on initiation, progression, and maintenance in some types of neoplasia that include lung and breast cancer [16,17]. In contrast, in other cell types, such as ovarian cells, knock-in of mutated, constitutively-active p110α PI3K resulted in cell hyperplasia, but not invasive cancer. Nevertheless, concomitant *PTEN* deletion led to the development of serous ovarian adenocarcinoma [18]. These findings suggest that in some cell types, PTEN loss may cooperate with other genetic alterations to induce cancer development. Furthermore, germline *PTEN* mutations are responsible for the rare, cancer-prone syndromes collectively referred to as PTEN hamartoma tumor syndrome, characterized by various benign and malignant tumors (breast, endometrial, thyroid, renal, and colon) [19].

PTEN has PI3K-independent functions, being involved in genome stability, chromatin remodeling, and double-strand DNA breaks’ repair [12], although the molecular mechanisms that regulate these functions are far from being fully understood [20]. Nevertheless, *PTEN* anomalies have been shown to contribute to endometrial carcinogenesis through defective DNA repair [21].

PTEN could also act as a protein phosphatase, as it is capable of dephosphorylating both phospho-serine/threonine and phospho-tyrosine residues [22]. Focal adhesion kinase (FAK) is one of the few PTEN protein substrates that have been identified [23].

In recent years, three additional PTEN variants have been identified: PTEN-long [24], PTENα [25], and PTENβ [26]. PTEN-long is secreted outside the cell, can be detected in human plasma, and plays an important role in the control of PTEN-induced putative kinase protein 1 (PINK1)-catalyzed phosphorylation of ubiquitin [27]. PTENα regulates mitochondrial functions and energy metabolism [25], as well as neutrophil chemotaxis [28], while PTENβ localizes predominantly to the nucleolus where it associates with and dephosphorylates nucleolin, thereby acting as a negative regulator of rDNA transcription [26].

Although the roles of PTEN as a tumor suppressor have been extensively documented and are well established, accumulating evidence indicates that PTEN functions are of fundamental importance in regulating physiological processes in healthy HSCs [29] and intestinal stem cells [30], as well as in normal T-, B-, and NK-cells [31,32,33,34]. Checking PI3K signaling is particularly important, as excessive levels of PIP3 not only oppositely affect many of the same processes, but can also somehow transform cells (for example HSCs) and lead to cancer [35].

Regarding T-cells, by limiting the amount of PIP3 available within the cell, PTEN directly opposes the PI3K/Akt/mTOR axis, thereby influencing the selection of developing thymocytes, as well as the activation of mature T-lymphocytes. T-cells with uncontrolled PI3K/Akt/mTOR activity, as a result of PTEN loss, contribute to the development of both autoimmune disorders and lymphomas [36]. For instance, T-cell–selective deletion of *Pten* leads to a premalignant state in the CD4^+^CD8^+^ double-positive thymocyte population that is followed by development of CD4^+^ T-cell lymphomas in secondary lymphoid organs, including the lymph nodes and the spleen [37,38,39].

PTEN is often inactivated via different mechanisms in human T-ALL, where it can be associated with chemotherapy and targeted therapy resistance, as well as a poor prognosis [40].

In this review, we discuss how PTEN-loss-of-function drives and sustains T-ALL via the activation of multiple signaling pathways. A better knowledge of the regulation of these networks could be of fundamental importance in their exploitation for an improved therapy and outcome of T-ALL.

## 2. PTEN and T-ALL Development in Mice

The observation that *Pten^−/−^* mice showed an embryonic lethal phenotype [41] prompted several groups to develop conditional knockout models, where *Pten* was selectively targeted in HSCs/hematopoietic progenitor cells/T-cells [42].

### 2.1. PTEN Conditional Deletion in HSCs

Several studies have demonstrated that *Pten* loss in HSCs/hematopoietic progenitor cells invariably causes T-ALL development (although with a different penetrance, depending on the model used) that is preceded by a myeloproliferative disorder [35,43,44,45]. In particular, Guo and coworkers [44] showed that *Pten* conditional deletion in murine HSCs from fetal liver led to a T-ALL where self-renewable leukemia initiating cells (LICs) were enriched in the c-Kit^mid^/CD3^+^/Lin^−^ bone marrow (BM) compartment. These LICs were capable of driving T-ALL development in four serial cohorts of mice and displayed significantly increased unphosphorylated β-catenin levels that sustained their renewal. Importantly, when one of the alleles of the β-catenin gene was ablated, the incidence of T-ALL caused by the loss of *Pten* substantially decreased, while leukemia progression was delayed in those mice that still developed the disorder. However, it should be emphasized here that treatment of this Pten-null T-ALL model with PI3K inhibitors was effective prior to the onset of leukemia, but not after leukemia was already underway [46,47]. This observation suggests that *Pten* ablation is not responsible for maintaining LSC stemness features, once LICs have been generated. In this context, it is important to underline that recent findings have highlighted how self-renewal of T-ALL LICs is driven and maintained by β-catenin via a spleen focus-forming virus proviral integration oncogene 1 (SPI1)/hepatitis A virus cellular receptor 2 (HAVCR2) regulatory circuit [48] (see later on in this article). T-ALL LICs displayed a chromosomal translocation involving *T-cell receptor α/δ and c-Myc* (*Tcrα/δ-c-Myc*), which resulted in aberrant overexpression of the avian myelocytomatosis viral homolog (c-MYC) oncogene [44]. These findings are extremely interesting as *c-MYC* translocations involving the T-cell receptor (TCR) loci have been identified in a small (~3%) subset of T-ALL patients displaying poor prognostic markers [49]. Importantly, the T-ALL LICs identified by Guo and coworkers [44] could be eradicated by co-targeting with selective inhibitors the deregulated pathways driven by PI3K and c-MYC [47].

Remarkably, at variance with adult stem cells, *Pten* deletion did not activate PI3K signaling or promote leukemogenesis in neonatal HSCs, thereby indicating that developmental stage dictates the tumor-suppressive functions of PTEN [50]. Miething et al. [51] exploited a transgenic mouse model where *Pten* expression in HSCs was regulated in both a time- and tissue-specific manner, to show that PTEN loss in the postnatal period sensitized T-ALL cells to C-C chemokine ligand 25 (CCL25), which is mainly expressed in mucosal epithelial cells in the small intestine. By interacting with its receptor C-C chemokine receptor 9 (CCR9, which was found to be highly expressed on *Pten*-null leukemic cells), CCL25 drove leukemic cell infiltration into the intestine, but not into other organs, such as the liver or the spleen [51]. Therefore, also signals originating from the microenvironment could strongly influence PTEN-loss-induced leukemogenesis, by producing a form of intratumoral heterogeneity.

PTEN loss characterizes a murine model of T-ALL where leukemogenesis was induced by overexpression of constitutively-active tropomyosin-related kinase A (ΔTRKA) or of TRKB/brain-derived neurotrophic factor (BDNF, the TRKB ligand) in Lin^−^/Sca1^+^/c-Kit^+^ HSCs [52]. During clonal evolution, these T-ALL cells acquired activating mutations in neurogenic locus notch homolog protein 1 (NOTCH1) and lost PTEN function due to loss of either one or both *Pten* alleles. Moreover, *Pten* inactivating mutations in exons 5 and 6 were detected in some clones. These findings show that PTEN loss can also be a secondary genetic lesion acquired during T-ALL clonal evolution.

Bornschein and coworkers [53] took advantage of a recently-developed murine pro-T-cell culture system [54] for unraveling the mechanisms underlying the cooperation between TAL1 transcription factor overexpression and *Pten* deletion, i.e., a combination of abnormalities frequently observed in T-ALL patients (see further on in this review) [55,56]. This system was used to demonstrate that the cooperation between TAL1 overexpression and *Pten* deletion resulted in an upregulated stem cell-like gene signature and increased E2 transcription factor (E2f) signaling activity and metabolic reprogramming characterized by higher lactate levels due to an increased flux of glucose into the tricarboxylic acid cycle [53]. Surprisingly, TAL1 overexpression per se was a growth disadvantage to pro-T-cells that could be, however, relieved by *Pten* deletion that induced Akt activation and expression of c-MYC and E2f. These findings emphasize how the signaling networks of healthy T-cells can be altered and/or taken over by the oncogenic events that characterize T-ALL.

### 2.2. PTEN Conditional Deletions in T-Cells

The use of transgenic murine cell lines where *Cre* recombinase is under the control of T-cell-specific promoters allowed several groups to demonstrate that the developmental stage of T-cells is critical for the outcome of *Pten* loss. *Pten* ablation in the double-negative thymocyte population led to the development of T-cell lymphomas that caused the death of the animals within 10–17 weeks. [37,39,57,58]. Nevertheless, T-cell lymphomas were also observed when *Pten* was ablated at later stages of thymocyte development, by using the CD4-*Cre* system [38]. Very recently, it was shown that human primary T-ALL cells expressing αβTCR are frequently deficient for PTEN and fail to respond strongly to TCR activation [59]. It is well known that signaling through αβTCR is a fundamental determinant of thymocyte fate and can result in two opposite outcomes during T-cell development: cell death or survival/differentiation [60]. However, the exact roles played by TCR in the transformation of developing T-cells remain to be defined [61]. Using *Pten*-null T-ALL murine models, it was shown that abrogation of TCR accelerated cancer development, whereas the overexpression of a fully-functional [62] transgenic αβTCR led to the onset of TCR-negative lymphomas while delaying tumorigenesis [59]. This group also demonstrated that pre-neoplastic *Pten*-null thymocytes with a fully-functional αβTCR underwent early clonal deletion and did not progress to neoplastic cells. In contrast, cells with a non-functional αβTCR that are normally deleted in the positive selection process in the thymus passed selection and developed T-ALL. Altogether, these findings document that a fully-functional αβTCR signaling suppresses leukemogenesis caused by *Pten* ablation and suggest that PTEN likely plays a fundamental role during positive selection [59]. In contrast, another group had previously shown that the loss of γcTCR inhibited thymic exit of *Pten*-deficient T-cells [63].

On the other hand, *Pten* deletion did not cause the development of T-cell lymphomas in either CD4^+^ helper T-cells or peripheral T-cells [64,65]. The aforementioned results unequivocally demonstrate that neoplastic transformation occurs only if *Pten* is deleted during thymocyte development, but not in mature, peripheral T-lymphocytes, thereby supporting the hypothesis that the tumor suppressor functions of PTEN are tightly dependent on the developmental stage of T-cells.

### 2.3. Studies in Human T-ALL Cells

The findings obtained with murine models have been partially confirmed when primary human T-ALL cells of a patient who relapsed and displayed a *PTEN* deletion at relapse, but not at diagnosis, were xenografted in mice. PTEN knockdown via shRNA in leukemic cells obtained at diagnosis conferred a selective advantage in competitive xenotransplantation experiments. These findings hint that PTEN loss in human T-ALL could promote the expansion of leukemic clones endowed with upregulated LIC activity, although in this case, *PTEN* deletion could not be the initial driving force for T-ALL development [66]. More recently, PTEN loss has been analyzed in human T-ALL samples displaying the stem cell leukemia (SCL)/TAL1 interrupting locus (STIL) protein/TAL1 fusion product [67] that were xenografted in mice and compared with specimens collected at diagnosis [68]. PTEN loss was detected as a secondary event in some subclones of leukemic cells.

Overall, these two studies seem to suggest that PTEN loss in human T-ALL is unlikely to be a founder or truncal mutation, but rather part of additional genomic lesions that may be critical for increased malignancy, as well as for clonal selection of the leukemic cells. It is worth emphasizing here that this experimental approach reproduces quite faithfully the process of leukemia progression from diagnosis toward relapse.

## 3. PTEN Regulation in Human T-ALL

Over the years, it has become clear that both genetic and non-genetic mechanisms lead to PTEN-loss-of-function in human T-ALL cells.

### 3.1. Genetic Mechanisms of PTEN Inactivation

*PTEN*-loss-of-function is due to predominantly monoallelic point mutations, gene deletions, or micro-deletions [4,55,69,70,71,72,73,74,75,76,77,78,79]. The frequency of *PTEN* alterations is variable, most likely due to differences in patient cohort size and/or methodology used; however, they have been detected in 11–27% of childhood T-ALL cases and 5–17% of adult patients. In children, *PTEN* mutations were mostly identified in *TAL/LMO* patients, although the association was not significant, while the incidence of mutations was significantly lower in the *TLX* cluster [55,71]. In adults, PTEN abnormalities were more frequently detected in younger, TCR-positive, STIL/TAL1-positive, NOTCH1/F-box WD repeat-containing protein 7 (FBXW7) unmutated patients with high leukemic bulk tumors [72].

Point mutations almost invariably affect exon 7 of *PTEN* that partially encodes the C2-domain [69,70]. Interestingly, these mutations seem to be specific to human T-ALL, as in other types of cancer, the hotspot for mutations is at exon 5, which encodes the phosphatase domain of PTEN [80]. Point mutations at exon 7 lead to carboxy terminal truncation of PTEN and are associated with reduced or lost PTEN expression in T-ALL patients [71,72], as truncated PTEN undergoes rapid degradation [81] via the ubiquitin-mediated proteasomal pathway [82].

The phosphatase domain-coding exons (2–6) display heterogeneously-sized deletions in about 3–8% of pediatric and adult T-ALL patients. These deletions affect almost exclusively exon 2 [69,71,72]. Furthermore, a detailed analysis of a large cohort of childhood T-ALL patients has led to the discovery of microdeletions, originating from illegitimate recombination events (see later in this article), in exons 2/3 and 4/5 [74]. Such microdeletions were detected in about 8% of patients.

Interestingly, when *PTEN* alterations were studied in a pediatric cohort, 67% of the cases displayed more than one *PTEN* anomaly, with some patients having up to four alterations [75]. These findings suggest the existence of multiple leukemic subclones displaying various PTEN anomalies, with each of these subsets possibly having different biological and clinical features.

### 3.2. Non-Genetic Mechanisms of PTEN Inactivation

Aberrant splicing of *PTEN* transcripts has been rarely detected in both pediatric [71] and adult [83] T-ALL patients. The exact origin of these defective events remains to be determined, as there were no mutations found in the first 20–30 intronic bases that flanked the splice donor/acceptor sites of the affected exons. However, defective splicing resulted in abolished PTEN expression [71].

PTEN is negatively regulated at the transcriptional level via aberrant NOTCH1 signaling, one of the major regulatory pathways of growth and metabolism in T-ALL cells [84]. The NOTCH1 signaling downstream target, hairy and enhancer of split-1 (HES-1), represses PTEN transcription by occupying regulatory sequences of the *PTEN* promoter [83].

In cancer cells, PTEN levels can be downregulated either epigenetically or post-transcriptionally by non-coding RNAs, including microRNAs (miRs) [85]. In murine T-ALL cells, miR-19 represses PTEN expression [86]. Although this oncogenic miR is highly expressed in human T-ALL cells [87], its ability to downregulate PTEN in this setting has not been demonstrated yet. Other miRs identified in human T-ALL and potentially targeting PTEN [88,89] include miR-20a and miR-92 [90], miR-148 [91], and miR-20b-5p and miR-363-3 [79]. Therefore, all of these miRs might contribute to the pathophysiology of T-ALL via downregulation of PTEN.

In T-ALL cells, PTEN protein is inactivated via posttranslational mechanisms that include both oxidation by reactive oxygen species (ROS) [92] and phosphorylation by casein kinase 2 (CK2) [92,93]. In particular, CK2 phosphorylates a cluster of serine/threonine residues (Ser^370^/Ser^380^/Thr^382^/Thr^383^/Ser^385^) located at the C-tail of the PTEN molecule [94,95]. Ser^370^/Ser^385^ are the most important residues for CK2-mediated downregulation of PTEN activity towards its substrate, PIP3 [96]. This results in higher PIP3 levels and PI3K/Akt activation [92].

## 4. Oncogenetic Functions of PTEN in T-ALL Cells

### 4.1. PI3K-Dependent Functions

**Figure 1:**
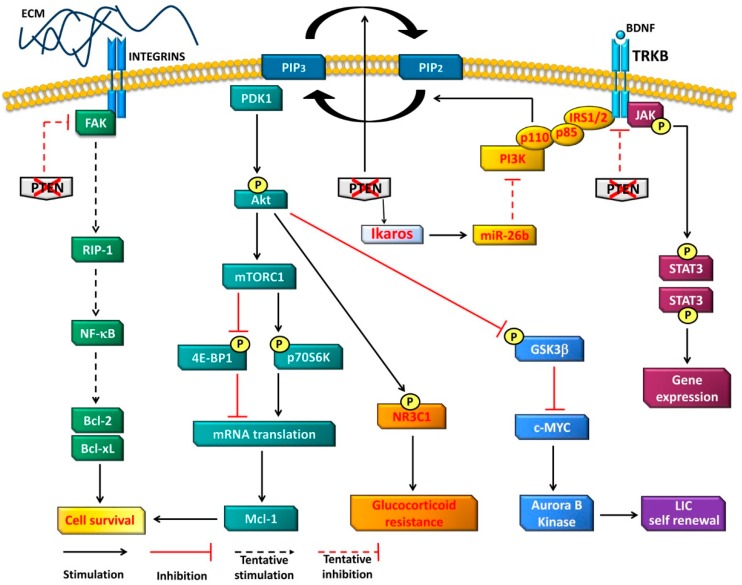
Main oncogenetic networks upregulated in PTEN-deficient T-ALL cells. For details, see the main text.

PTEN loss-induced leukemogenesis has been mostly related to the activation of the PI3K/Akt/mTOR network (Figure 1). Indeed, upregulated PI3K/Akt/mTOR signaling has been consistently observed in human T-ALL specimens with *PTEN* mutations and/or deletions [72,83,97]. Moreover, several lines of evidence indicate that this signaling pathway is critically involved in the development of T-cell malignant disorders that follow PTEN loss in mouse models [98,99,100,101]. In particular, regarding PI3K, it has been shown that the p110γ and p110δ PI3K catalytic subunits cooperatively sustain leukemogenesis in *Pten*-mutant mice [101]. Of note, recent findings have demonstrated that PTEN represses PI3K/Akt signaling also via an Ikaros transcription factor/miR-26b axis that directly downregulates the expression of *PIK3CD*, i.e., the gene encoding the p110δ PI3K catalytic subunit [102] (Figure 1).

Nevertheless, the precise roles played by Akt activation downstream of PTEN loss in mice are still awaiting to be fully elucidated as, surprisingly, phosphoinositide-dependent kinase 1 (PDK1)/Akt signaling was not required for the proliferation of *Pten*-null thymocytes [99]. Consistently, expression of constitutively-active Akt in transgenic mouse thymocytes did not impact on their cell cycle distribution in vivo [103]. It might be that PDK1/Akt signaling is implicated in processes different from cell proliferation, such as thymocyte migration to secondary lymphoid organs through the control of chemokines and adhesion receptors expressed by PTEN-null cells [99].

Nevertheless, by activating Akt, PTEN loss downregulates glycogen synthase kinase 3β (GS3Kβ) activity, thereby preventing c-MYC degradation mediated by GS3Kβ [104]. Accordingly, there is an inverse correlation between c-MYC expression levels and PTEN expression in samples from T-ALL patients [105]. In mice, c-MYC activates aurora kinase B, which is somehow involved in the regulation of self-renewal of *Pten*-null LICs [47] (Figure 1). However, the regulation and maintenance of the key determinants of stemness in T-ALL LICs is a complex phenomenon. For instance, in a recent paper, Zhu et al. [48] identified SPI1 as a master regulator of LIC stemness in a murine *Pten*-null T-ALL model. This group demonstrated that *Spi1* expression is initiated by the PTEN-controlled β-catenin overactivation characterizing murine *Pten*-null T-ALL. However, both *Spi1* expression and LIC stemness were reinforced and maintained via a β-catenin-SPI1-HAVCR2 regulatory circuit independently of the leukemogenic driver mutation. Altering any component of this circuit either genetically or pharmacologically prevented LIC formation or eliminated existing LICs, whereas inhibition of the PI3K/Akt pathway had little effect on the LIC number. LICs lost their stem cell features when *Spi1* expression was silenced by DNA methylation; however, *Spi1* expression could be reactivated by treatment with the DNA methyltransferase inhibitor, 5-azacytidine. Of note, similar regulatory circuitries could be also detected in human T-ALL cells [48].

It is worth highlighting here that in humans, *SPI1* encodes the PU-box 1 protein (PU.1), one of the most important members of the E-twenty-six (Ets) transcription factor family. PU.1 plays a broad range of roles in hematopoiesis, including the early stages of T-cell development [106], where its overexpression induces progenitor T-cell proliferation while blocking differentiation [107,108]. Importantly, gene fusions involving *SPI-1* were detected in ~4% of 181 pediatric T-ALL cases displaying a characteristic gene expression profile and a uniformly poor overall survival [108].

LIC self-renewal also requires mTOR [46,47]. mTOR acts as the catalytic subunit of two distinct multiprotein complexes, known as mTOR complex 1 (mTORC1) and mTOR complex 2 (mTORC2) [109,110]. Of these, the first one is probably involved in regulating LIC self-renewal, as leukemic clones with upregulated LIC activity displayed increased mTORC1 activity in a T-ALL zebrafish model [111]. Nevertheless, both mTORC1 and mTORC2 are likely involved in T-ALL development, as ablation of either raptor (an mTORC1 component) or rictor (an mTORC2 component) increased the survival of *Pten*-mutant mice in a significant manner [50,112]. mTORC1 controls key cellular functions that include mRNA translation, metabolism, and autophagy, while mTORC2 is mainly involved in cell survival and motility [113,114,115,116]. mRNA translation sustains T-cell development in vivo, as, when ribosome function was reduced in *Pten*-mutant mice by expressing a mutated ribosome protein L24 (Rpl24), protein synthesis was downregulated, thereby decreasing leukemogenesis [117]. mTORC1 upregulates mRNA translation mainly through the phosphorylation of the translation initiation component eukaryotic translation initiation factor 4E-binding protein 1 (4E-BP1) and 70-kDa ribosomal protein S6 kinase (p70S6K) [118] (Figure 1). Both 4E-BP1 and p70S6K seem to play a role in T-ALL, although p70SK deficiency in *Pten*-null HSCs only slightly delayed leukemia initiation [52,119].

### 4.2. PI3K-Independent Functions

The existence of a protein phosphatase activity of PTEN is highly controversial; however, PTEN could restrain FAK activity by dephosphorylation [23,120]. Upregulated FAK activity was observed in both *Pten*-deleted murine thymocytes and human PTEN-null T-ALL cells [121], where it elicited survival signals via a receptor activating protein 1 (RIP1)/nuclear factor-κB (NF-κB)/B-cell lymphoma-extra-large (Bcl-xL)/B-cell lymphoma 2 (Bcl-2) signaling axis [122] (Figure 1). Moreover, when FAK was deleted in *Pten*-mutated hematopoietic progenitor cells, T-ALL development was significantly delayed. Interestingly, it was demonstrated that FAK was activated via interactions with the extracellular matrix (ECM) and integrins in *PTEN*-mutated T-ALL cells (Figure 1). Consistent with the existence of two parallel signaling cascades leading to survival of leukemic cells, treatment with FAK pharmacological inhibitors increased the sensitivity of murine and human PTEN-null T-ALL cells to drugs targeting the PI3K/AKT/mTOR pathway, both in vitro and in vivo [121]. Although it is still unclear how PTEN could downregulate FAK activity, these results indicate that both the PI3K-dependent and -independent functions of PTEN are necessary to dampen T-cell malignant transformation.

### 4.3. Genomic Stability

**Figure 2:**
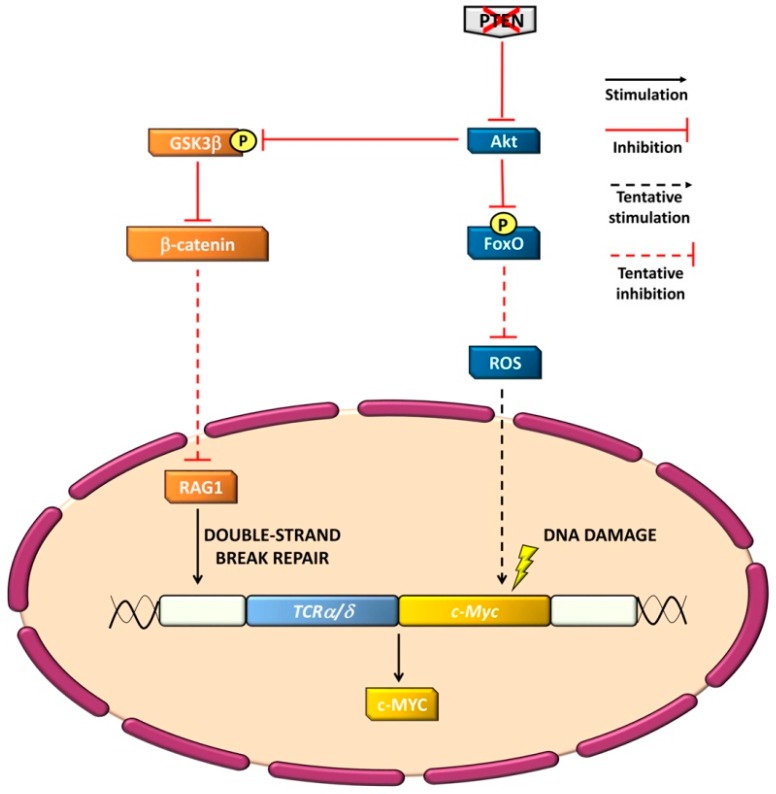
Genomic instability as a consequence of PTEN-loss-of activity in T-ALL cells. When PTEN activity is lost, active Akt phosphorylates and inhibits both GSK3β and the FoxO family of transcription factors. Inhibition of GSK3β leads to β-catenin activation and downregulation of double-strand break DNA repair mediated by RAG1. Moreover, upregulated ROS levels, due to decreased FoxO transcription factor activity, determine DNA damage. As a consequence, the typical *Tcrα/δ-c-Myc* chromosomal translocation occurs, resulting in increased expression of c-MYC.

PTEN has been defined as a guardian of the genome [20,123]. As previously highlighted in this article, *Pten* ablation in murine HSCs or developing T-cells leads to chromosomal translocations between the *Tcrα/δ* locus and the *c-Myc* oncogene. This observation suggests that PTEN may oppose genomic instability [38,44,46,51]. In solid tumors, this function of PTEN is independent of its phosphatase activity and requires PTEN translocation to the nucleus [20]. However, in T-ALL, PTEN controls genome stability most likely via the activation of phosphatase-dependent cytosolic signaling networks, as demonstrated by a study from Newton and coworkers [124]. This group developed an elegant murine model where a phosphatase-dead PTEN-mutant form was overexpressed in T-cells that lacked wild-type PTEN. As a consequence, the mice developed a CD4^+^ T-cell lymphoma displaying the typical *Tcrα/δ-c-Myc* chromosomal translocation. Although these findings demonstrate that PTEN phosphatase activity is required to prevent genomic instability, the molecular basis underlying this process remains unclear. Interestingly, thymocytes expressing the phosphatase-dead PTEN mutant upregulated the Akt/forkhead box protein O (FoxO) axis and displayed a FoxO-dependent upregulation of ROS levels that might contribute to DNA damage [125]. In this context, it is worth emphasizing that active FoxO transcription factors counteract ROS generation by increasing the levels of antioxidant enzymes [126,127]. However, once phosphorylated by Akt, FoxOs are no longer active [128]; hence, ROS production could increase (Figure 2).

The genomic instability that characterizes *Pten*-null cells may also originate from an aberrant activation of β-catenin, as β-catenin upregulation prevented the repair of recombination activating gene (RAG)-mediated DNA double-strand breaks in murine thymocytes, thus leading to *Tcrα-δ/c-Myc* translocations and T-cell lymphoma development [129] (Figure 2). The activation of β-catenin in the context of PTEN-loss-of-function could be due to enhanced PI3K/Akt signaling and subsequent downregulation of GSK3β, which is a well-known repressor of β-catenin [130] (Figure 2).

Moreover, if RAG1 recombinase was knocked out in *Pten*-mutated HSCs, *Tcrα/δ-c-Myc* translocations, LIC formation, and T-ALL development were blocked. These observations indicate that the loss of *Pten* induces a genomic instability that is critical for T-cell malignant transformation [46]. Of note, also the PTEN microdeletions identified in some pediatric T-ALL cases by Mendes and coworkers [74] occur as a consequence of aberrant RAG-mediated recombination events.

## 5. Clinical Impact of PTEN Loss on T-ALL Patient Outcome

Several groups have addressed the prognostic impact of PTEN loss in a number of pediatric and adult T-ALL patient cohorts [4,69,70,71,72,73,75,77,78,131,132,133]. In general, PTEN anomalies have been associated with poor response to chemotherapy, increased risk of relapse, and adverse long-term outcome in all of the studies except for three that were performed in childhood patient cohorts [75,131,132]. Interestingly, the unfavorable clinical effects of inactivating PTEN mutations in pediatric T-ALL patients could be neutralized by coexisting NOTCH1 activating mutations [73]. Another study that analyzed a large cohort of 573 pediatric and adult T-ALL patients treated with the GRAALL03/05 and FRALLE2000 therapeutic protocols came to the intriguing conclusion that the prognostic impact of PTEN loss is likely dependent on the underlying type of genetic anomaly [77]. In particular, while large deletions predicted lower five-year overall survival and disease-free survival both in children and in adults, mutations could not be associated with a worse prognosis. These findings may indicate that a detailed analysis of the type of genetic anomaly would be useful to refine risk stratification based on *PTEN* status. However, in the most recently-published study, all types of *PTEN* abnormalities were found to be significantly linked to an unfavorable prognosis in a cohort of 162 T-ALL pediatric cases treated with the ALL IC-BFM 2002 and 2009 therapeutic protocols [133]. Therefore, despite many efforts, the clinical impact of *PTEN* alterations is still an area of intense debate and likely depends on the methods of analysis, as well as on the treatment context and the presence of other genetic anomalies.

## 6. Therapeutic Perspectives

Glucocorticoids (GCs) represent an important component of the armamentarium we have at our disposal for treatment of T-ALL and, in combination with other chemotherapeutic agents, contribute to ~80% of pediatric patients achieving long-term overall survival [134]. Early response to GCs predicts a positive prognosis, while patients who do not show a robust response relapse more frequently and experience a negative outcome [135]. Although the molecular basis underlying GC resistance in T-ALL cells remains to be defined, several lines of evidence indicate the PI3K/Akt/mTOR axis as one of the signaling networks involved in GC-resistance [111,136,137]. Active Akt impairs GC-induced gene expression via the phosphorylation of the GC receptor, nuclear receptor subfamily 3 group C member 1 (NR3C1), at Ser^134^. This phosphorylation blocks GC-dependent translocation of NR3C1 to the nucleus [97] (Figure 1). The selective Akt inhibitor, MK-2206, restored GC-induced NR3C1 nuclear translocation and reversed the response of PTEN-null T-ALL cells to GCs in vitro and in vivo. Of the three Akt isoforms expressed in mammalian cells (Akt1, Akt2, Akt3), Akt2 seems to be the most relevant to GC-resistance, as it was highly expressed in GC-resistant PTEN-null human T-ALL cell lines. Importantly, GC-resistance could be reversed in vitro and in vivo by CCT128930, a highly specific Akt2 inhibitor [138]. The pro-apoptotic protein Bcl-2-like protein 11 (Bim) is one of the effectors of GC-mediated cytotoxicity, as its upregulated expression via Akt2 inhibition increased GC-sensitivity [138]. Similar results had been previously reported by an independent group that used the dual PI3K/mTOR inhibitor NVP-BEZ235 to counteract GC-resistance in PTEN-null human T-ALL cells [139].

Regarding innovative therapies, numerous preclinical studies have demonstrated that inhibitors selectively blocking PI3K activation are promising drugs for T-ALL treatment [140]. In PTEN-null models of T-ALL, the dual p110γ/δ PI3K inhibitor, CAL-130, prolonged survival in an animal model. Moreover, the drug blocked proliferation and activated proapoptotic signaling pathways in human T-ALL cell lines and primary samples [101]. However, these findings could not be confirmed by other groups that showed that inhibition of all four isoforms of the p110 PI3K catalytic subunit was more effective than dual p110γ/δ PI3K inhibition in inducing cytotoxicity in human PTEN-null T-ALL cells [141,142].

This discrepancy may partly depend on the fact that in human PTEN-null T-ALL, TRKB collaborates with PTEN loss by activating both the PI3K/Akt and Janus kinase (JAK)/signal transducer and activator of transcription 3 (STAT3) pathway. In particular, it was demonstrated that p110α and p110δ are the major PI3K isoforms that mediate PI3K/Akt signaling evoked by TRKB activation in PTEN-null T-ALL cells [143]. These findings are consistent with observations of other groups showing that TRKB promotes survival and metastasis of lung adenocarcinoma, breast cancer, and chondrosarcoma by upregulating PI3K/Akt and JAK/STAT3 signaling [144,145,146]. TRKB could be activated in PTEN-null T-ALLs by an autocrine loop involving BDNF [52]. In neurons, PI3K does not directly bind TRKB [147,148]. However, when activated by its ligand BDNF, TRKB auto-phosphorylates tyrosine residues in its juxta membrane domain. These residues are the docking sites for a wide variety of adaptor molecules, including insulin receptor substrate (IRS)-1 and -2, which increase PI3K/Akt activity [147] (Figure 1). Regarding JAK/STAT3 upregulation, it could also depend, at least in part, on secretion of cytokines that is increased in cells lacking PTEN [143]. Interestingly, PI3K inhibitors alone had little effect on STAT3 phosphorylation in T-ALL cells, suggesting that JAK/STAT3 activation might drive a partial resistance to PI3K inhibitors. Accordingly, a combined treatment consisting of PI3K and STAT3 inhibitors resulted in maximal suppression of T-ALL cell proliferation and tumor progression in vivo. Of note, TRK family receptors are rapidly emerging as potential therapeutic targets in various cancer types [149,150], and TRK inhibitors are being tested in phase I/II trials [151,152].

The NOTCH1 signaling pathway represents an attractive target to treat T-ALL, given that NOTCH1 is activated by mutations in 65–70% of T-ALL patients [153] and is a central driver of T-ALL cell survival, growth, and metabolism [8]. NOTCH1 signaling can be effectively targeted by γ-secretase inhibitors (GSIs) [154]. *Pten* loss in murine NOTCH1-mutated T-ALL cells drives an oncogenic addiction to PI3K/Akt, which sustains leukemic cell growth independently of NOTCH1 signaling, thereby causing resistance to GSI treatment [155]. In a murine model, *Pten* deletion activated the expression of multiple genes involved in ribosomal RNA processing, amino acid, and nucleotide synthesis, likely due to upregulation of mTORC1 activity. Expression of these genes is normally repressed in response to treatment with GSIs. Moreover, *Pten* loss relieved the block of glycolysis and glutaminolysis induced by GSIs, a phenotype that could be mimicked by overexpression of a constitutively active form of Akt [155]. Clinical trials with GSIs have so far given disappointing results in T-ALL patients, due to several issues [156,157]. However, in light of the previously-reported findings, future NOTCH1-targeted therapies might be more efficacious if GSIs could be combined with either PI3K or Akt inhibitors [40].

Rapamycin, an mTORC1 inhibitor, has proven its efficacy to eradicate LICs in PTEN-null preclinical models of murine and human T-ALL, when combined with either the aurora kinase inhibitor, VX-680, or the c-Myc inhibitor, JQ1 [47]. mTORC1 inhibitors might be useful also in the context of PTEN-null T-ALLs treated with the Bcl-2 inhibitor navitoclax (ABT-263), as upregulated mTORC1 increases the expression levels of myeloid cell leukemia sequence 1 (Mcl-1), thereby mitigating responses to ABT-263 [158,159] (Figure 1).

## 7. Conclusions

The evidence gained from the use of knockout models supports the hypothesis that PTEN-loss-of-function in murine models plays important roles in T-ALL initiation and development. Similar evidence is still lacking for human T-ALL, although PTEN anomalies seem to be important for clonal evolution and disease progression. Nevertheless, PTEN loss is crucial to the emergence of drug-resistance, thereby negatively influencing patient outcome. Our understanding of the signaling pathways upregulated by PTEN-loss-of-function is growing quite rapidly. Therefore, a deeper knowledge of the complex oncogenic networks activated by PTEN alterations should provide promising targeted treatments for improving T-ALL prognosis.

## Abbreviations

Bcl-xL	B-cell lymphoma-extra large
Bcl-2	B-cell lymphoma 2
Bim	Bcl-2-like protein 11
BM	Bone marrow
BDNF	Brain-derived neurotrophic factor
CCL25	C-C chemokine ligand 25
CCR9	C-C chemokine receptor 9
CK2	Casein kinase 2
c-MYC	Avian myelocytomatosis viral homolog
E2f	E2 transcription factor
ECM	Extracellular matrix
ERK	Extracellular signal-regulated kinase
ETP T-ALL	Early T-cell precursor T-ALL
Ets	E-twenty-six
4E-BP1	Eukaryotic translation initiation factor 4E-binding protein 1
FAK	Focal adhesion kinase
FBXW7	F-box WD repeat-containing protein 7
FLT3	Fms-related tyrosine kinase3
FoxO	Forkhead box protein O
GCs	Glucocorticoids
GSI	γ-secretase inhibitors
GS3Kβ	Glycogen synthase kinase 3β
HAVCR2	Hepatitis A virus cellular receptor 2
HES-1	Hairy and enhancer of split-1
HOX	Homeobox
HSCs	Hematopoietic stem cells
JAK	Janus kinase
IRS	Insulin receptor substrate
LMO	LIM domain only
Mcl-1	Myeloid cell leukemia sequence 1
MEK	Mitogen-activated protein kinase kinase
miRs	MicroRNAs
MRE11	Meiotic recombination 11 homolog 1
mTOR	Mechanistic target of rapamycin
mTORC1	mTOR complex 1
mTORC2	mTOR complex 2
NF-κB	Nuclear factor-kB
NOTCH1	Neurogenic locus notch homolog protein 1
NF-κB	Nuclear factor kappa B
NR3C1	Nuclear receptor subfamily 3 group C member 1
PDK1	Phosphoinositide-dependent kinase 1
PI3K	Phosphatidylinositol-3 kinase
PINK-1	PTEN-induced putative kinase protein 1
PIP2	Phosphatidylinositol(4,5)P2
PIP3	Phosphatidylinositol(3,4,5)P3
PTEN	Phosphatase and Tensin homolog, deleted on chromosome TEN
PU.1	PU-box 1 protein
p70S6K	70-kDa ribosomal protein S6 kinase
RAD50	Rad1 homolog 50
RAF	Rapidly-accelerated fibrosarcoma
RAG	Recombination activating gene
RAS	Rat sarcoma
RIP1	Receptor activating protein 1
Rpl24	Ribosome protein L24
ROS	Reactive oxygen species
SCL	Stem cell leukemia
STIL	SCL/TAL1 interrupting locus
SPI1	Spleen focus forming virus proviral integration oncogene 1
STAT3	Signal transducer and activator of transcription 3
T-ALL	T-cell acute lymphoblastic leukemia
TAL1	T-cell acute lymphocytic leukemia 1
TCR	T-cell receptor
Tcrα/δ-c-Myc	T-cell receptor α/δ-c-Myc
TLX	Nuclear receptor subfamily 2 group E member
TRK	Tropomyosin-related kinase A
